# Evidence for the Rhythmic Perceptual Sampling of Auditory Scenes

**DOI:** 10.3389/fnhum.2019.00249

**Published:** 2019-07-23

**Authors:** Christoph Kayser

**Affiliations:** Department for Cognitive Neuroscience & Cognitive Interaction Technology, Center of Excellence, Bielefeld University, Bielefeld, Germany

**Keywords:** hearing, auditory perception, rhythmic perception, reverse correlation, perceptual weights, delta band, theta band

## Abstract

Converging results suggest that perception is controlled by rhythmic processes in the brain. In the auditory domain, neuroimaging studies show that the perception of sounds is shaped by rhythmic activity prior to the stimulus, and electrophysiological recordings have linked delta and theta band activity to the functioning of individual neurons. These results have promoted theories of rhythmic modes of listening and generally suggest that the perceptually relevant encoding of acoustic information is structured by rhythmic processes along auditory pathways. A prediction from this perspective—which so far has not been tested—is that such rhythmic processes also shape how acoustic information is combined over time to judge extended soundscapes. The present study was designed to directly test this prediction. Human participants judged the overall change in perceived frequency content in temporally extended (1.2–1.8 s) soundscapes, while the perceptual use of the available sensory evidence was quantified using psychophysical reverse correlation. Model-based analysis of individual participant’s perceptual weights revealed a rich temporal structure, including linear trends, a U-shaped profile tied to the overall stimulus duration, and importantly, rhythmic components at the time scale of 1–2 Hz. The collective evidence found here across four versions of the experiment supports the notion that rhythmic processes operating on the delta time scale structure how perception samples temporally extended acoustic scenes.

## Introduction

Perception seems to be systematically controlled by rhythmic processes in the brain (VanRullen, 2016; Haegens and Zion Golumbic, 2018; Helfrich, 2018). These rhythmic processes may for example reflect the excitability sensory neurons (Lakatos et al., 2005; Romei et al., 2008; Kayser et al., 2015), the selection of specific features for a behavioral response (Wyart et al., 2012; Wostmann et al., 2016), or the attentional modulation of perception (Busch et al., 2009; Busch and VanRullen, 2010). The perceptually-relevant rhythmic brain activity not only manifests in systematic relations between brain and behavior, such as better perceptual detection rates following a specific pattern of brain activity (Ng et al., 2012; Henry et al., 2014; Iemi and Busch, 2018), but can also reflect directly in behavioral data: for example, reaction times or perceptual accuracies in visual detection tasks are modulated at time scales of theta (~4 Hz) and alpha (~8 Hz) band activity (Fiebelkorn et al., 2011; VanRullen and Dubois, 2011; Landau and Fries, 2012; Song et al., 2014). In the case of hearing, neuroimaging studies have similarly shown that pre-stimulus delta (~1 Hz) and theta activity (~4 Hz) determine whether a sound is detected or influence how it is perceived (Ng et al., 2012; Henry et al., 2014, 2016; Strauss et al., 2015; ten Oever and Sack, 2015; Kayser et al., 2016). As in vision, the influence of rhythmic activity manifests also in behavioral data (Barnes and Jones, 2000; Hickok et al., 2015; Ho et al., 2017). In general, the apparent influence of rhythmic neural activity on behavior has been linked to rhythmic modes of perception, which facilitate the amplification of specific, e.g., expected, stimuli and mediate the alignment of endogenous neural activity to the regularities of structured sounds such as speech (Schroeder and Lakatos, 2009; Giraud and Poeppel, 2012). Indeed, the time scales of human perceptual sensitivity and the time scales at which rhythmic auditory activity shapes perception seem to be well matched (Edwards and Chang, 2013; Keitel et al., 2018).

While it remains unclear whether truly spontaneous brain activity affects auditory perception (VanRullen et al., 2014; Zoefel and VanRullen, 2017), it is clear that once the auditory system is driven by sounds rhythmic activity becomes engaged and shapes perception (Henry et al., 2014; Zoefel and VanRullen, 2015, 2017; Lakatos et al., 2016; Haegens and Zion Golumbic, 2018). Still, many studies linking neural activity and auditory percepts have relied on brief acoustic targets, such as tones (Ng et al., 2012; Kayser et al., 2016; McNair et al., 2019), gaps in noise (Henry and Obleser, 2012; Henry et al., 2014), or isolated words or syllables (Strauss et al., 2015; ten Oever and Sack, 2015). Yet, a key prediction from models postulating a rhythmic mode of perception (Schroeder and Lakatos, 2009; Zoefel and VanRullen, 2017; Haegens and Zion Golumbic, 2018), and from models linking cortical delta activity with sensory gain (Kayser et al., 2015; Iemi and Busch, 2018), is that this rhythmic activity should also shape how sensory information is integrated over time: the perceptual weighting of temporally dispersed acoustic information should be modulated at precisely those time scales at which neural activity has been shown to shape the detection or perception of brief and isolated sounds. Note that this precise hypothesis is distinct from the roles of rhythmic activity highlighted by work on the relevance of the pseudo-rhythmic structure of speech for comprehension (Rosen, 1992; Ghitza and Greenberg, 2009; Zoefel et al., 2015), by studies using rhythmic electric brain stimulation to enhance speech comprehension (Zoefel and Davis, 2017; Wilsch et al., 2018), or studies showing that various aspects of acoustic and linguistic information are represented in (pseudo-) rhythmic brain activity (Di Liberto et al., 2015; Daube et al., 2019; Yi et al., 2019).

**Figure 1:**
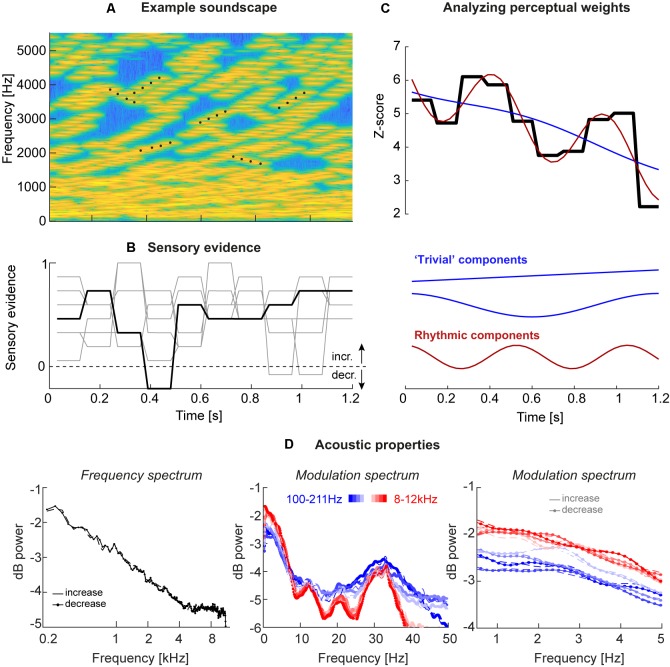
Acoustic stimuli and analysis. **(A)** Stimuli consisted of “soundscapes” consisting of 30 four-tone sequences either in- or de-creasing in frequency (example sequences are marked by black dots). The fraction of sequences moving in the same direction changed randomly across trials and between “epochs” of a specific duration, which varied between experiments (see Table 1). **(B)** Each trial was characterized by the level of motion evidence for the soundscape to in- or de-crease, with the evidence being independent between epochs (periods of constant evidence) and trials, and varying around a participant-specific threshold. The black line presents the evidence for the soundscape shown in panel **(A)**, the gray lines the evidence for other trials, all with (on average) increasing frequency. An evidence of 1 correspond to a fully coherent soundscape, evidence of 0 to a completely random soundscape (15 tone sequences increasing, 15 decreasing). **(C)** The trial-averaged single participant perceptual weights (average sensory evidence for trials where participants responded with “up” or “down,” combined after correcting the sign of down responses) were analyzed using regression models. These models distinguished trivial temporal structure arising from linear trends or U/V shaped profiles locked to stimulus duration (blue) from rhythmic structure at faster time scales (red). The black graph displays the perceptual weight of one example participant together with the best-fitting trivial and rhythmic contributions. **(D)** Acoustic properties of these soundscapes, shown here for Experiment 3. Upper panel: frequency spectrum revealing an approximate 1/f structure. Middle and lower panels: temporal modulation spectra, derived as the frequency spectrum of band-limited envelopes at different frequencies (color-coded). The middle panel reveals a peak at 33 Hz, the duration of individual tones. The right panel shows the lack of specific modulation peaks at the behaviorally relevant range between 1 Hz and 5 Hz, as well as a lack of difference between soundscapes with in- and de-creasing frequency content. All spectra are averaged across all trials and participants (*n* = 20; Experiment 3).

We here tested this prediction directly at the level of behavior (Kayser, 2019). That is, we asked whether the perceptual use of acoustic information available in a continuous and extended (1 s or longer) stimulus is structured rhythmically at the delta/theta band time scale. To this end, we employed an acoustic variant of the frequently used visual random dot motion stimulus (Mulder et al., 2013). In our study, participants judged the overall direction of change in the perceived frequency content of soundscapes composed of a dense sequence of random tones, a specific fraction of which systematically in- or de-creased in pitch (Figure 1A). The level of sensory evidence available in each trial about the direction of frequency change was sampled independently in epochs of between 90 ms to 180 ms (in different versions of the task) allowing us to quantify the influence of the moment by moment varying acoustic evidence on participant’s judgments (Figure 1B). Across four versions of this experiment, we found consistent evidence that the perceptual sampling of temporally extended sounds is structured by processes operating at the time scale of around 1–2 Hz.

## Materials and Methods

We report the data obtained from a total of 79 volunteers (age: 19–28 years, 67% female). The data was collected following written informed consent and briefing about the purpose of the study. Participants received either monetary compensation or course credits. All had self-reported normal hearing. The study was conducted in accordance with the Declaration of Helsinki and was approved by the local ethics committee of Bielefeld University. The required sample size per experiment was set* a priori* to at least *n* = 20 based on recommendations for behavioral studies (Simmons et al., 2011). For two of the experiments, an number of 23 was collected as data collection proceeded partly in parallel. Seven participants that had participant in Experiment 1 also participated in Experiment 3. Additional data from 18 participants were collected but not analyzed, as the data did not comprise sufficiently many trials (see data exclusion below).

### Acoustic Stimuli

Stimuli were presented *via* headphones (Sennheiser HD200 Pro) at an average intensity of 65 dB root mean square level (calibrated using a Model 2250 sound level meter; Bruel & Kjær, Denmark) while participants were seated in an acoustically shielded room. Stimulus presentation was controlled from Matlab (Mathworks) using routines from the Psychophysics toolbox (Brainard, 1997).

The acoustic stimuli (“soundscapes”) consisted of 30 simultaneous sequences of pure tones (each tone had 30 ms duration, 6 ms on/off cosine ramps; zero inter-tone interval except the cosine ramp) that either increased or decreased in frequency and each sequence lasted four tones (see Figure 1A for a spectro-temporal representation). The initial frequency of each sequence was drawn independently (uniform between 128 Hz and 16,384 Hz), and each sequence in/decreased in steps of 20 cents. To construct a specific soundscape, each tone sequence started at a random position within the four-tone sequence, so that the start time of each sequence was independent of that of all others (hence all sequences had the same “life-time,” but started at a random “age”). Also, the precise onset times of individual tones were jittered between sequences by (uniformly) up to 30 ms, to ensure that individual tones did not all present at the same time. Once a sequence reached the 4th tone, it was replaced by a new sequence starting at a random frequency. The impression of an overall in- or decrease in frequency over time was manipulated by changing the fraction of sequences that in- or de-creased. This fraction was coded as between 0 and 1, with 0.5 indicating half the sequences as in- and the other half as decreasing, and 1 indicating that all sequences increased. Each trial was characterized by the intended direction of change (in- or decrease) and the respective level of “motion evidence” at which this direction was expressed. Here motion evidence is defined as the deviation from ambiguous evidence (that is, 0.5). The relative motion evidence ranges from 0 (random) to 1 (fully coherent) and was used to characterize the task difficulty, and to quantify participant’s perceptual thresholds (see Figure 1B for example traces).

To quantify the perceptual use of the acoustic information at different time points by individual participants, this motion evidence was manipulated 2-folds. First, for each participant, we determined (in an initial block) the participant specific perceptual threshold of motion evidence required to achieve around 80% correct performance (García-Pérez, 1998). To this end, participants performed the task based on trials with the task difficulty being determined following three interleaved 1-up 2-down staircases, each starting at a different level of difficulty (range 0.15–0.8), and using multiplicative reduction of step sizes. The threshold for each staircase was obtained from an average of six reversals, excluding the initial four reversals. The participant’s threshold was then derived as the mean of the three individual thresholds.

Second, we manipulated the motion evidence between trials, and over time within a trial, around this subject-specific threshold. To this end, we sub-divided each soundscape into “epochs” and randomly and independently sampled the motion evidence from a Gaussian distribution around the participant-specific threshold (SD of 0.15 or 0.25, depending on the experiment). The duration of these epochs varied between experiments from 90 ms to 180 ms (see Figure 1B for examples; see Table 1). Practically, for a given trial, it was first determined whether the soundscape should in- or decrease. Then, the epoch-specific levels of motion evidence were drawn and then the sequences of individual tones were generated as described above. Thereby, the direction of sweep of each tone sequence could change at the start of a new epoch, where the directions of change of all sequences were re-drawn randomly to meet the momentary level of motion evidence. The total duration of each soundscape varied between 1,200 ms and 1,800 ms (Table 1). Each experiment consisted of 800 trials per participant. Inter-trials intervals lasted 800 ms to 1,200 ms (uniformly). For technical reasons, in some of the earlier Experiments (1 and 2) it had not been enforced that participants could respond only after the end of the soundscape, leading to premature responses. We hence imposed a minimal number of 750 valid trials for a participant to be included and we excluded data from 18 participants for Experiments 1 and 2 for this technical reason. Based on this criterion, we analyzed the data of *n* = 23 participants for Experiments 1 and 2, and *n* = 20 each for Experiments 3 and 4 (Table 1), whereby seven participants performed by Experiments 1 and 3.

**Table 1:** Parameters and results for each experiment, including the duration of sound scape and the “epochs” over which the sensory evidence changed randomly, the number of epochs (sampling frequency) over which the perceptual weights were determined (Df), the number of participants (N), the best frequency determined by each model criterion (Fpeak) and the relative model criterion vs. the best trivial model.

	Soundscape	Epoch	*Df*	*N*	*F*peak cv-AICc	Δcv-AICc	*F*peak WAIC	ΔWAIC
Experiment 1	1,800 ms	180 ms (5.5 Hz)	10	23	1.6 Hz	183	1.6 Hz	186
Experiment 2	1,600 ms	90 ms (11 Hz)	17	23	1.3 Hz	32	1.3 Hz	71
Experiment 3	1,200 ms	120 ms (8.3 Hz)	10	20	2 Hz	121	2 Hz	135
Experiment 4	1,700 ms	120 ms (8.3 Hz)	14	20	1.2 Hz	13	2 Hz	46

### Statistical Properties of the Acoustic Stimuli

Given the possibility that temporal structure of the acoustic envelope may shape the perceptual sampling of these soundscapes, we computed the temporal modulation spectrum in different frequency bands. To this end, we first computed the band-limited Hilbert envelope of each soundscape in 10 logarithmically spaced bands between 100 Hz and 12 kHz (3rd order Butterworth filter; Chandrasekaran and Ghazanfar, 2009). We then computed the average temporal modulation (frequency-) spectrum for each band across soundscapes and participants, separately for trials with in- or de-creasing frequency content (Figure 1D).

### Response Templates

**Figure 2:**
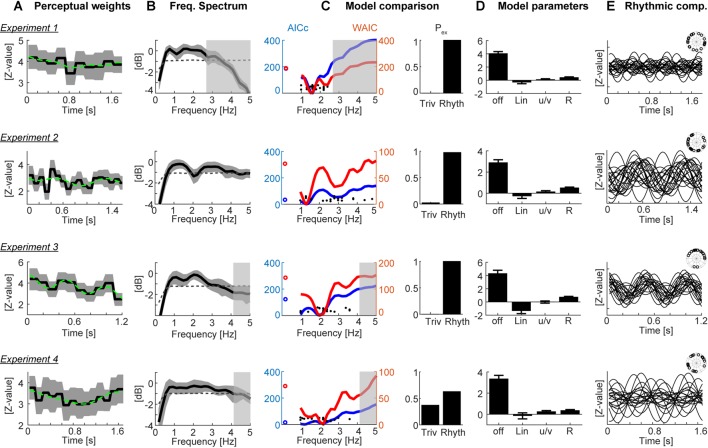
Results. **(A)** Participant-averaged perceptual weights (black solid) with group-level two-sided 5% bootstrap confidence intervals (gray area) and the best-fitting model (green dashed). Units are in z-scores relative to a within-participant bootstrap baseline. **(B)** Participant-averaged frequency spectrum of the perceptual weights (black solid), with two-sided 5% bootstrap confidence interval (gray area). The dashed line represents the average spectrum obtained after time-shuffling the weights. **(C)** Model comparison results. The left curves show the group-level cv-AICc (blue; left y-axis) and watanabe-akaike information criterion (WAIC; red; right y-axis) values for the best trivial model (open circles) and frequency dependent models. Individual participant’s best frequencies are denoted by solid black dots. The bars on the right show the exceedance probabilities of a comparison between the trivial model and the rhythmic model derived at the frequency yielding the lowest group-level cv-AICc. **(D)** Model parameters (betas) of the trivial contributions (offset, linear slope, u/v shaped profile) and the rhythmic component (R, root-mean-squared amplitude of sine and cosine components). Bars and error-bars indicate the group-level mean and SEM. **(E)** Rhythmic component of the best-fitting model for each participant (lines) and phase of this component each participant (inset). In panels **(B,C)** the gray transparent boxes black out those frequency ranges which cannot be faithfully reconstructed given the experiment specific epoch duration (i.e., behavioral sampling rate); hence only the clear regions are meaningful.

In the domain of motion evidence, each trial consists of a sequence of statistically independent samples of evidence for the direction of change in the soundscape. Hence, in this domain one can use the epoch by epoch evidence in a psychophysical reverse correlation procedure (Marmarelis, 1978; Eckstein and Ahumada, 2002). To compute perceptual weights (also known as response templates), trials were split according to direction of change and participants’ responses. Then response-specific averages of the motion evidence were computed and were converted to units of z-scores within each participant using bootstrapping: the actual weights were standardized relative to the distribution of 4,000 sets of surrogate weights (Neri and Heeger, 2002; Chauvin et al., 2005). These weights indicate how strongly the acoustic evidence at each moment influences the perceptual judgments, with zero indicating no influence and positive values indicating that the in- (de)creases in the stimulus were rated as in- (de)creasing by the participant (see Figure 2A). To visualize the spectral composition of these templates we computed their power spectrum after standardizing the overall signal power (Figure 2B). It should be noted that this calculation makes the assumption that the perceptual weight, and any temporal structure therein, is consistent across trials within a participant, as the reverse correlation tries to assign a fixed (relatively high or low) weight to each epoch. Please note that due to the different duration of the epochs across experiments, the range of frequencies at which rhythmic patterns in behavioral can be recovered vary (2.75, 5.5, 4.15, and 4.15 Hz, respectively).

### Analysis of Rhythmic Components in Response Templates

The main goal of the analysis was to determine whether the participant-specific weights were characterized by a significant rhythmic component (see Figure 1C for example weights). To this end an analysis was implemented that first selectively extracted non-rhythmic structure, such as: (i) the overall offset; (ii) a linear in- or de-crease over time; and (iii) a U/V shaped profile time-locked to the stimulus duration (modeled as cos(2*pi*t*F_exp_), with F_exp_ = 1/stimulus duration). For each participant’s perceptual template, we computed regression models comprising a collection of the non-rhythmic patterns (only i, i+ii, and i+ii+iii) and selected from these best fitting model based on the group-level AICc (see below). We then extended this “trivial” model by a rhythmic contribution modeled at frequencies varying between 1 Hz and 5 Hz, comprising both sine- and cosine components of the same frequency. The results were derived and are shown over a fixed frequency range, but the actually recoverable time scales differ between experiments, as the epoch duration, and hence the effective sampling rate of the perceptual weights differed. The interpretable range is indicated for each experiment in Figure 2.

Formal model-comparison was then used to determine, at the group-level, whether there was evidence for the rhythmic model to better explain the data than the trivial model (Burnham and Anderson, 2004; Gelman et al., 2014; Palminteri et al., 2017). That is, we tested whether the addition of a rhythmic component, e.g., at the delta band time scale, helped to explain more variance in the perceptual weights than models not featuring this rhythmic component. The comparison was based on the log-likelihoods computed for each participant’s data and regression model. It is important to note that the inclusion of both sine and cosine components in the rhythmic model allowed for each participant to have a potentially different phase-alignment to the stimulus (see Figure 2E for the resulting best rhythmic components per participant).

Two recommended approaches for model comparison were used to compare the evidence in favor of each model, taking into account that the rhythmic model has additional degrees of freedom and may trivially explain the data better than the non-rhythmic model (Burnham and Anderson, 2004; Gelman et al., 2014; these additional degrees of freedom arise from the sine and cosine components). First, regression models were fit using a Monte-Carlo approach to compute the Watanabe-Akaike information criterion (WAIC), which captures the out-of-sample predictive power when penalizing each model (Gelman et al., 2014). This calculation was implemented using the Bayesian regression package for Matlab (Makalic and Schmidt, 2016), using 10,000 samples, 10,000 burn-in samples and a thinning factor of 5. Second, regression models were compared using a cross-validated version of the corrected Akaike criterion (cv-AICc; Hurvich and Tsai, 1991). Response templates were fit using half the data and the log-likelihood of each model was computed on the other half; the AICc was then averaged over 10 independent two-fold CV runs. Group-level comparison was performed by computing the group-level WAIC and cv-AICc, and by computing the exceedance probabilities of each model based on −0.5*cv-AICc [implemented using VBA toolbox in Matlab (Rigoux et al., 2014)].

To determine whether the selection of a specific frequency-dependent model over the trivial model was indeed specific to the behavioral data, and was not induced by any other factor in the analysis such as the temporal binning (see e.g., Vorberg and Schwarz, 1987) for pitfalls involved in testing reaction times for rhythmic patterns), we repeated the model comparison using randomized data (Zoefel et al., 2019). We randomly paired stimuli and responses and computed the probability of selecting the rhythmic model (at the group-level frequency determined using the original data) over the trivial model across 2000 instances of randomized data based on the cv-AICc.

The specific approach of contrasting the predictive power of different regression models, rather than e.g., testing the significance of specific regression parameters (here, of the sine and cosine terms of the rhythmic component), was chosen for a number of reasons. First, the use of cross-validation and Monte-Carlo methods allows capturing the out-of-sample predictive power of each model, while the obtained regression coefficients reflect only with specific-sample effect (Burnham and Anderson, 2004; Gelman et al., 2014). Second, a test on the regression parameters would need to consider the magnitude of the combined sine/cosine component, as the phase could differ between participants. This would require a somewhat problematic one-sided test on strictly positive values; and third, quantifying the predictive power of individual models allows a direct side by side comparison of the contribution of different rhythmic time scales to behavior, over and above the predictive power offered by a model without such a rhythmic component (that is, the respective AIC differences in Figure 2C).

## Results

Participants judged the perceived direction of frequency change in soundscapes constructed based on randomly varying task-relevant evidence. Across four variants of the experiment, the stimulus duration ranged from 1,200 to 1,800 ms, while the time scale at which perceptual weights were sampled varied between 5.5 Hz and 11 Hz (Table 1). These time scales were chosen* a priori* to allow capturing potential rhythmicity at time scales between 1 Hz and 10 Hz, as deemed relevant by a large body of neurophysiological and neuroimaging studies, and the combinations of soundscape and epoch duration were chosen to reflect of combinations of time scales. The frequency spectrum of these soundscapes followed largely a 1/f structure (Figure 1D) while the temporal modulation spectra of band-limited envelopes revealed no specific spectral peaks in the low-frequency range of interest, and no clear differences between directions of frequency change, that could have been exploited for behavior (1–5 Hz; Figure 1D).

Figure 2A displays the group-averaged perceptual weights for each experiment. The weights were significant for all time points (based on the 5% percentile of a bootstrap distribution). This is in line with the task difficulty being set to be around each participant’s perceptual threshold and indicates that participants in large used the evidence from all time points to solve the task. For each dataset, the group-level weights exhibited a rich structure, comprising linear trends and a U/V shaped profile tied to the duration of each soundscape. To determine the contribution of such “trivial,” i.e., non-rhythmic, contributions we fit three candidate models to each participant’s data. Group-level model comparison revealed that the model featuring all three trivial factors (offset, slope, U/V profile) better explained the data than a reduced model: the group-level ΔAICc of the full vs. the reduced model were 211.2, 4.6, 93.3, 117.2 for Experiments 1–4, respectively, and the group-level exceedance probabilities of the full model were p_ex_ = 0.76, 0.49, 0.99, and 0.94. Only for Experiment 2 there was no clear evidence for any of the models to explain the data better than the others.

We then used the best trivial model (determined at the group-level, separately for each experiment) to quantify the extent to which the addition of a rhythmic contribution helped to better explain the perceptual weights. The prominence of temporal structure at the time scale between 1 Hz and 3 Hz is also highlighted by the frequency spectra in Figure 2B. Formal model comparison between the best trivial model and the frequency dependent models revealed that the addition of a rhythmic component between 1.2 Hz and 2 Hz significantly improved the model fit, even when taking into account the increased degrees of freedom (Figure 2C). The time scales best explaining the perceptual data were 1.6 Hz, 1.3 Hz, 2 Hz and 1.2 Hz based on the cv-AICc for the four experiments, respectively (Table 1). When using the WAIC, we found the same frequencies, except for Experiment 4 (here WAIC identified 2 Hz as best frequency). Both the cv-AICc and the WAIC model criteria identified the rhythmic model (defined at the group-level AICc-based best frequency) as significantly better than the trivial model for each dataset: the Δcv-AICc values of best rhythmic over the trivial model were 183, 32, 121, 13, respectively, the ΔWAIC values were 186, 71, 135, and 46 [with values >30 usually considered as very strong evidence in favor of one model (Burnham and Anderson, 2004)]. To further substantiate this result, we obtained group-level exceedance probabilities of the best rhythmic model in comparison to the trivial model: for three out of four experiments these clearly favored the rhythmic model: p_ex_ = 1, 0.97, 1, 0.62 for Experiments 1–4 (Figure 2C).

Given that this apparent rhythmic structure may also emerge simply as byproduct of sub-sampling the behavioral sensitivity at a fixed time scale, we repeated the model fitting after shuffling behavioral responses across trials (Zoefel et al., 2019). We computed the probability that the model incorporating the best group-level frequency derived from the original data better explained the data than the trivial model in the shuffled data (based on the cv-AICc): these probabilities were small and revealed the actual effects as (close-to) significant: *p* = 0.08, 0.076, 0.040, and 0.068 for Experiments 1–4, respectively.

To visualize the best models, Figure 2D displays the model parameters for the best-fitting rhythmic model, while Figure 2E displays the rhythmic component for each individual participant. In particular, for Experiment 3 the data reveal a clear alignment of perceptual weights across participants.

Closer inspection of Figure 2C shows that the WAIC reveals two local minima for several of the experiments: besides the overall best model at frequencies between 1.2 Hz and 2 Hz, also rhythmic components at frequencies between 2 Hz and 4 Hz better explain the actual data than the trivial model. The precise frequency of this second component varied between experiments (Experiment 1: 2.4 Hz, ΔWAIC = 123 vs. trivial model; Experiment 2: 2.8 Hz ΔWAIC = 43; Experiment 3: 3.4 Hz ΔWAIC = 36; Experiment 4: 3.8 Hz ΔWAIC = 24). This observation suggests that effectively multiple rhythmic components may underlie auditory perception.

## Discussion

We investigated whether the relation between the sensory evidence contained in temporally extended soundscapes and participant’s judgments is governed by rhythmic components, as predicted by theories of rhythmic modes of listening, as well as studies linking delta/theta band neural activity with perception. The four experiments differed in the overall stimulus duration (Table 1; 1,200 ms to 1,800 ms) and the time scale at which perceptual weights were sampled (5.5–11 Hz). Despite these variations in the experimental paradigm, we found converging evidence that the perceptual sensitivity profiles contain relevant rhythmic structure at the time scales between 1.2 Hz and 2 Hz. That the rhythmic models indeed better explain the perceptual use of acoustic information than a trivial model only containing linear and U/V shaped trends is supported by the use of two criteria for formal model comparison and the comparison of the original and shuffled data. Importantly, the soundscapes used in the experiment did not exhibit obvious spectral structure at these behaviorally relevant times scales (see Figure 1D).

The perceptual weights featured pronounced non-rhythmic temporal structure, such as linear trends (e.g., Experiment 3, Figure 2A) or a U-shaped profile emphasizing early and late stimulus components (e.g., Experiment 4). Such stimulus-locked temporal sensitivity is frequently observed in perceptual decision-making paradigms and in part may reflect the participant-specific strategies for analyzing the sensory environment, temporal leakage in decision processes, or the urgency to respond (Okazawa et al., 2018; Waskom et al., 2018). Importantly, our results show that this sensitivity profile is augmented by a more rapidly changing temporal structure that emerges at precisely those time scales deemed relevant for auditory perceptual sensitivity by neuroimaging studies. Consistently across the four experiments, the best rhythmic models featured a perceptual sensitivity that was modulated with a frequency between 1.2 Hz and 2 Hz.

Previous work has shown that auditory cortical delta band activity is tied to changes in the network state related to an overall rhythmic fluctuation in neural background activity, visible both in spontaneous and acoustically driven states (Lakatos et al., 2005; Kayser et al., 2015; Guo et al., 2017). In particular strong engagement of auditory delta band activity has been implied in acoustic filtering of attended information and the task-relevant engagement of auditory networks (Lakatos et al., 2013, 2016; O’Connell et al., 2014) and plays a central role in theories of rhythmic modes of listening (Schroeder and Lakatos, 2009; Zoefel and VanRullen, 2017; Haegens and Zion Golumbic, 2018). While electrophysiological studies reporting behaviorally-relevant rhythmic patterns of brain activity often identified frequencies in the theta band as important (Henry and Obleser, 2012; Ng et al., 2012; Henry et al., 2014; Kayser et al., 2016) some of these have identified multiple mechanisms operating at different time scales, including the delta band between 1 Hz and 2 Hz (Henry et al., 2014, 2016; McNair et al., 2019). Our results corroborate the behavioral relevance of neural mechanisms operating in the delta band for auditory perception and provide evidence for the potential existence of distinct and possibly multiplexed rhythmic mechanisms. One potential interpretation of the results is that a specific listening mode is triggered by the onset of the soundscape and engages rhythmic processes that are phase-aligned across trials, but possibly engage distinct optimal phases across individuals (Henry et al., 2014; Haegens and Zion Golumbic, 2018). A related question, which only future studies can address, is whether the relative importance of rhythmic processes is stronger shortly following the onset of each soundscape, or is equally important throughout the entire soundscape.

An intriguing question is whether the precise time scale of sensory sampling is fixed, at least within a participant, or whether it adapts to the momentary statistics of the relevant sounds. Across the four experiments, we found a considerable variation in the best sampling frequency for each individual participant (see Figure 2C), and in the resulting group-level frequencies (1.6 Hz, 1.3 Hz, 2 Hz and 1.2 Hz, respectively, based on the cv-AICc), suggesting that these can vary across a considerable range. Is it possible that the sampling frequency is shaped by the experimental context, such as the duration of the soundscapes within each experiment? Only a systematic, and within-participant, manipulation of this duration can address this. In the present data, the ratio of sound duration to sampling time scales ranged from 2.04 to 2.8. This could be taken as evidence against a fixed alignment of sampling frequency to stimulus duration, and rather speaks in favor of more idiosyncratic mechanisms. A related question is whether the contribution of different time scales to rhythmic perceptual sampling is shaped by the overall spectral or temporal modulation statistics of the stimulus (see Figure 1D). Again, a systematic manipulation of such sound properties is required to address whether e.g., the reduced weight of higher sampling frequencies is related to the reduced temporal modulation energy at these frequencies. It seems unlikely, but cannot be fully excluded, that perceptual sampling actually operates at a fast time scale than the epoch-based manipulation of sensory evidence, and simply is seen between 1 Hz and 2 Hz as a result of aliasing (the perceptual weights were effectively sampled at frequencies between 2.75 Hz and 5.5 Hz, depending on the experiment). Hence, any behavioral sampling occurring in the alpha band (about 8–12 Hz), such as known from visual or auditory spatial attention, may effectively be seen at lower frequencies in the present data (Landau and Fries, 2012; VanRullen and Macdonald, 2012; Wostmann et al., 2016).

Still, there are a few caveats to note. First, while the converging evidence across the four experiments is convincing, for each individual experiment the statistical likelihood of the rhythmic model to better explain the data than the trivial model in comparison to randomized data was only marginally significant. One possibility is that the estimated perceptual weights are noisy and more trials per participant would be required to obtain fully reliable estimates. Second, it could be that the preferred perceptual sampling frequency differs across participants, precluding a reliable estimate of a common group-level model. Indeed, the single-participant data reveal a considerable variability in their best-frequency (see Figure 2C). However, without the assumption of a fixed group-level frequency, it becomes difficult to determine whether a frequency dependent model fits the data significantly better than a null model. Third, and along similar lines, the present analysis makes the assumption that the time course of these weights is consistent across trials. Such a consistency may not be warranted, but rather the trial-specific sampling may be aligned to trial-to-trial variable neural processes (Ng et al., 2012; Henry et al., 2014). Only the inclusion of neuroimaging in future studies can dissociate these possibilities. Fourth, the presented analysis implicitly assumes that the participants made use of the full available acoustic information and used all tone sequences equally for their judgments. The perceptual reverse correlation procedure was implemented in the domain of the overall motion-evidence, based on which the different tone sequences were randomly assembled. Performing a reverse correlation in the full time-frequency domain would likely require much higher trial numbers as the degrees of freedom for the perceptual weights would increase considerably. As a result, participant specific biases towards particularly low or high sound frequencies may have reduced the power of the present analysis. Considering the degrees of freedom of the analysis, that is the number of effective weights per perceptual template, Table 1 reveals that the two experiments with the lowest number of free parameters were those yielding the larges evidence in favor of a rhythmic model, regardless of the total duration of the soundscape. This observation fits with the possibility that when sampling perceptual weights at finer temporal resolution or over additional dimensions, such as sound frequency, more trials would be required to obtain reliable estimates.

Also, the present results leave it unclear whether the rhythmic process(es) operate at the level of sensory encoding or decision making. When combined with fixed-duration stimuli, psychophysical reverse correlation cannot dissociate sensory from decision processes (Okazawa et al., 2018). While electrophysiological studies have directly demonstrated the relevance of auditory cortical delta band activity for neural sound encoding (O’Connell et al., 2014; Kayser et al., 2015) and perception (Lakatos et al., 2016), neuroimaging studies have shown that rhythmic brain activity may affect both the encoding of sensory information at shorter latencies and decision processes emerging later in frontal regions (Kayser et al., 2016; McNair et al., 2019). Work on visual decision making has also demonstrated the relevance of delta band activity for the accumulation of sensory evidence over time (Wyart et al., 2012). Hence it could be that the rhythmic patterns revealed here either reflect a change in the quality of the encoding of sensory evidence at each moment in time, which then results in a differential contribution to the participant’s judgment, or a direct change in the weight assigned during the accumulation of evidence for choice (Wyart et al., 2012; Drugowitsch et al., 2016). More work is required to better understand the interplay of rhythmic processes related to sensory encoding and of those related to the actual decision process.

To conclude, theories of rhythmic modes of listening, and neurophysiological data linking network activity to single neuron encoding, predict that rhythmic activity shapes how acoustic information is combined over time to judge extended soundscapes. The present study proposes one approach to test this and provides converging evidence in support of this prediction. Future work can capitalize on this approach to directly link electrophysiological signatures of rhythmic activity to the perceptual combination of acoustic information over time.

## Data Availability

The behavioral data and the required Matlab code for producing the stimuli, the analysis and figures are available from http://www.uni-bielefeld.de/biologie/cns/resources.html.

## Ethics Statement

The studies involving human participants were reviewed and approved by Ethics committee of Bielefeld University. The patients/participants provided their written informed consent to participate in this study.

## Author Contributions

CK conceived and implemented the study, analyzed the data, wrote the manuscript.

## Conflict of Interest Statement

The author declares that the research was conducted in the absence of any commercial or financial relationships that could be construed as a potential conflict of interest.

## References

[B1] Barnes R. Jones M. R. (2000). Expectancy, attention, and time. Cogn. Psychol. 41, 254–311. 10.1006/cogp.2000.0738 11032658

[B2] Brainard D. H. (1997). The psychophysics toolbox. Spat. Vis. 10, 433–436. 10.1163/156856897X00357 9176952

[B3] Burnham K. P. Anderson D. R. (2004). Multimodel inference: understanding AIC and BIC in model selection. Sociol. Methods Res. 33, 261–304. 10.1177/0049124104268644

[B5] Busch N. A. Dubois J. VanRullen R. (2009). The phase of ongoing EEG oscillations predicts visual perception. J. Neurosci. 29, 7869–7876. 10.1523/JNEUROSCI.0113-09.2009 19535598 PMC6665641

[B4] Busch N. A. VanRullen R. (2010). Spontaneous EEG oscillations reveal periodic sampling of visual attention. Proc. Natl. Acad. Sci. U S A 107, 16048–16053. 10.1073/pnas.1004801107 20805482 PMC2941320

[B6] Chandrasekaran C. Ghazanfar A. A. (2009). Different neural frequency bands integrate faces and voices differently in the superior temporal sulcus. J. Neurophysiol. 101, 773–788. 10.1152/jn.90843.2008 19036867 PMC2657063

[B7] Chauvin A. Worsley K. J. Schyns P. G. Arguin M. Gosselin F. (2005). Accurate statistical tests for smooth classification images. J. Vis. 5, 659–667. 10.1167/5.9.1 16356076

[B8] Daube C. Ince R. A. A. Gross J. (2019). Simple acoustic features can explain phoneme-based predictions of cortical responses to speech. Curr. Biol. 29, 1924.9–1937.9. 10.1016/j.cub.2019.04.067 31130454 PMC6584359

[B9] Di Liberto G. M. O’Sullivan J. A. Lalor E. C. (2015). Low-frequency cortical entrainment to speech reflects phoneme-level processing. Curr. Biol. 25, 2457–2465. 10.1016/j.cub.2015.08.030 26412129

[B10] Drugowitsch J. Wyart V. Devauchelle A. D. Koechlin E. (2016). Computational precision of mental inference as critical source of human choice suboptimality. Neuron 92, 1398–1411. 10.1016/j.neuron.2016.11.005 27916454

[B11] Eckstein M. P. Ahumada A. J. Jr. (2002). Classification images: a tool to analyze visual strategies. J. Vis. 2:1x. 10.1167/2.1.i 12678601

[B12] Edwards E. Chang E. F. (2013). Syllabic (approximately 2-5 Hz) and fluctuation (approximately 1–10 Hz) ranges in speech and auditory processing. Hear. Res. 305, 113–134. 10.1016/j.heares.2013.08.017 24035819 PMC3830943

[B13] Fiebelkorn I. C. Foxe J. J. Butler J. S. Mercier M. R. Snyder A. C. Molholm S. (2011). Ready, set, reset: stimulus-locked periodicity in behavioral performance demonstrates the consequences of cross-sensory phase reset. J. Neurosci. 31, 9971–9981. 10.1523/JNEUROSCI.1338-11.2011 21734288 PMC3343369

[B14] García-Pérez M. A. (1998). Forced-choice staircases with fixed step sizes: asymptotic and small-sample properties. Vision Res. 38, 1861–1881. 10.1016/s0042-6989(97)00340-4 9797963

[B15] Gelman A. Hwang J. Vehtari A. (2014). Understanding predictive information criteria for Bayesian models. Stat. Comput. 24, 997–1016. 10.1007/s11222-013-9416-2

[B16] Ghitza O. Greenberg S. (2009). On the possible role of brain rhythms in speech perception: intelligibility of time-compressed speech with periodic and aperiodic insertions of silence. Phonetica 66, 113–126. 10.1159/000208934 19390234

[B17] Giraud A. L. Poeppel D. (2012). Cortical oscillations and speech processing: emerging computational principles and operations. Nat. Neurosci. 15, 511–517. 10.1038/nn.3063 22426255 PMC4461038

[B18] Guo W. Clause A. R. Barth-Maron A. Polley D. B. (2017). A corticothalamic circuit for dynamic switching between feature detection and discrimination. Neuron 95, 180.5–194.5. 10.1016/j.neuron.2017.05.019 28625486 PMC5568886

[B19] Haegens S. Zion Golumbic E. (2018). Rhythmic facilitation of sensory processing: a critical review. Neurosci. Biobehav. Rev. 86, 150–165. 10.1016/j.neubiorev.2017.12.002 29223770

[B20] Helfrich R. F. (2018). The rhythmic nature of visual perception. J. Neurophysiol. 119, 1251–1253. 10.1152/jn.00810.2017 29357470

[B22] Henry M. J. Herrmann B. Obleser J. (2014). Entrained neural oscillations in multiple frequency bands comodulate behavior. Proc. Natl. Acad. Sci. U S A 111, 14935–14940. 10.1073/pnas.1408741111 25267634 PMC4205645

[B23] Henry M. J. Herrmann B. Obleser J. (2016). Neural microstates govern perception of auditory input without rhythmic structure. J. Neurosci. 36, 860–871. 10.1523/JNEUROSCI.2191-15.2016 26791216 PMC6601997

[B21] Henry M. J. Obleser J. (2012). Frequency modulation entrains slow neural oscillations and optimizes human listening behavior. Proc. Natl. Acad. Sci. U S A 109, 20095–20100. 10.1073/pnas.1213390109 23151506 PMC3523826

[B24] Hickok G. Farahbod H. Saberi K. (2015). The rhythm of perception: entrainment to acoustic rhythms induces subsequent perceptual oscillation. Psychol. Sci. 26, 1006–1013. 10.1177/0956797615576533 25968248 PMC4504793

[B25] Ho H. T. Leung J. Burr D. C. Alais D. Morrone M. C. (2017). Auditory sensitivity and decision criteria oscillate at different frequencies separately for the two ears. Curr. Biol. 27, 3643.e3–3649.e3. 10.1016/j.cub.2017.10.017 29153327

[B26] Hurvich C. M. Tsai C.-L. (1991). Bias of the corrected AIC criterion for underfitted regression and time series models. Biometrika 78, 499–509. 10.1093/biomet/78.3.499

[B27] Iemi L. Busch N. A. (2018). Moment-to-moment fluctuations in neuronal excitability bias subjective perception rather than strategic decision-making. eNeuro 5:ENEURO.0430-17.2018. 10.1523/eneuro.0430-17.2018 29911179 PMC6002263

[B28] Kayser C. (2019). Evidence for the rhythmic perceptual sampling of auditory scenes. bioRxiv [Preprint]. 10.1101/618652 PMC6663999 31396064

[B30] Kayser S. J. McNair S. W. Kayser C. (2016). Prestimulus influences on auditory perception from sensory representations and decision processes. Proc. Natl. Acad. Sci. U S A 113, 4842–4847. 10.1073/pnas.1524087113 27071110 PMC4855557

[B29] Kayser C. Wilson C. Safaai H. Sakata S. Panzeri S. (2015). Rhythmic auditory cortex activity at multiple timescales shapes stimulus-response gain and background firing. J. Neurosci. 35, 7750–7762. 10.1523/JNEUROSCI.0268-15.2015 25995464 PMC4438125

[B31] Keitel A. Gross J. Kayser C. (2018). Perceptually relevant speech tracking in auditory and motor cortex reflects distinct linguistic features. PLoS Biol. 16:e2004473. 10.1371/journal.pbio.2004473 29529019 PMC5864086

[B32] Lakatos P. Barczak A. Neymotin S. A. McGinnis T. Ross D. Javitt D. C.. (2016). Global dynamics of selective attention and its lapses in primary auditory cortex. Nat. Neurosci. 19, 1707–1717. 10.1038/nn.4386 27618311 PMC5127770

[B33] Lakatos P. Musacchia G. O’Connel M. N. Falchier A. Y. Javitt D. C. Schroeder C. E. (2013). The spectrotemporal filter mechanism of auditory selective attention. Neuron 77, 750–761. 10.1016/j.neuron.2012.11.034 23439126 PMC3583016

[B34] Lakatos P. Shah A. S. Knuth K. H. Ulbert I. Karmos G. Schroeder C. E. (2005). An oscillatory hierarchy controlling neuronal excitability and stimulus processing in the auditory cortex. J. Neurophysiol. 94, 1904–1911. 10.1152/jn.00263.2005 15901760

[B35] Landau A. N. Fries P. (2012). Attention samples stimuli rhythmically. Curr. Biol. 22, 1000–1004. 10.1016/j.cub.2012.03.054 22633805

[B36] Makalic E. Schmidt D. F. (2016). High-dimensional bayesian regularised regression with the bayesreg package. arXiv [Preprint]. Available online at: arxiv.org/abs/1611.06649

[B37] Marmarelis V. (1978). Analysis of Physiological Systems: The White-Noise Approach. New York, NY: Springer.

[B38] McNair S. W. Kayser S. J. Kayser C. (2019). Consistent pre-stimulus influences on auditory perception across the lifespan. Neuroimage 186, 22–32. 10.1016/j.neuroimage.2018.10.085 30391564 PMC6347568

[B39] Mulder M. J. Keuken M. C. van Maanen L. Boekel W. Forstmann B. U. Wagenmakers E. J. (2013). The speed and accuracy of perceptual decisions in a random-tone pitch task. Atten. Percept. Psychophys. 75, 1048–1058. 10.3758/s13414-013-0447-8 23572205 PMC3691469

[B40] Neri P. Heeger D. J. (2002). Spatiotemporal mechanisms for detecting and identifying image features in human vision. Nat. Neurosci. 5, 812–816. 10.1038/nn886 12101403

[B41] Ng B. S. Schroeder T. Kayser C. (2012). A precluding but not ensuring role of entrained low-frequency oscillations for auditory perception. J. Neurosci. 32, 12268–12276. 10.1523/JNEUROSCI.1877-12.2012 22933808 PMC6621531

[B42] O’Connell M. N. Barczak A. Schroeder C. E. Lakatos P. (2014). Layer specific sharpening of frequency tuning by selective attention in primary auditory cortex. J. Neurosci. 34, 16496–16508. 10.1523/JNEUROSCI.2055-14.2014 25471586 PMC4252556

[B43] Okazawa G. Sha L. Purcell B. A. Kiani R. (2018). Psychophysical reverse correlation reflects both sensory and decision-making processes. Nat. Commun. 9:3479. 10.1038/s41467-018-05797-y 30154467 PMC6113286

[B44] Palminteri S. Wyart V. Koechlin E. (2017). The importance of falsification in computational cognitive modeling. Trends Cogn. Sci. 21, 425–433. 10.1016/j.tics.2017.03.011 28476348

[B45] Rigoux L. Stephan K. E. Friston K. J. Daunizeau J. (2014). Bayesian model selection for group studies—revisited. Neuroimage 84, 971–985. 10.1016/j.neuroimage.2013.08.065 24018303

[B46] Romei V. Brodbeck V. Michel C. Amedi A. Pascual-Leone A. Thut G. (2008). Spontaneous fluctuations in posterior α-band EEG activity reflect variability in excitability of human visual areas. Cereb. Cortex 18, 2010–2018. 10.1093/cercor/bhm229 18093905 PMC2517102

[B47] Rosen S. (1992). Temporal information in speech: acoustic, auditory and linguistic aspects. Philos. Trans. R. Soc. Lond. B Biol. Sci. 336, 367–373. 10.1098/rstb.1992.0070 1354376

[B48] Schroeder C. E. Lakatos P. (2009). Low-frequency neuronal oscillations as instruments of sensory selection. Trends Neurosci. 32, 9–18. 10.1016/j.tins.2008.09.012 19012975 PMC2990947

[B49] Simmons J. P. Nelson L. D. Simonsohn U. (2011). False-positive psychology: undisclosed flexibility in data collection and analysis allows presenting anything as significant. Psychol. Sci. 22, 1359–1366. 10.1177/0956797611417632 22006061

[B50] Song K. Meng M. Chen L. Zhou K. Luo H. (2014). Behavioral oscillations in attention: rhythmic α pulses mediated through theta band. J. Neurosci. 34, 4837–4844. 10.1523/JNEUROSCI.4856-13.2014 24695703 PMC6802725

[B52] Strauss A. Henry M. J. Scharinger M. Obleser J. (2015). α phase determines successful lexical decision in noise. J. Neurosci. 35, 3256–3262. 10.1523/JNEUROSCI.3357-14.2015 25698760 PMC6605582

[B53] ten Oever S. Sack A. T. (2015). Oscillatory phase shapes syllable perception. Proc. Natl. Acad. Sci. U S A 112, 15833–15837. 10.1073/pnas.1517519112 26668393 PMC4702974

[B54] VanRullen R. (2016). Perceptual cycles. Trends Cogn. Sci. 20, 723–735. 10.1016/j.tics.2016.07.006 27567317

[B55] VanRullen R. Dubois J. (2011). The psychophysics of brain rhythms. Front. Psychol. 2:203. 10.3389/fpsyg.2011.00203 21904532 PMC3163286

[B56] VanRullen R. Macdonald J. S. (2012). Perceptual echoes at 10 Hz in the human brain. Curr. Biol. 22, 995–999. 10.1016/j.cub.2012.03.050 22560609

[B57] VanRullen R. Zoefel B. Ilhan B. (2014). On the cyclic nature of perception in vision versus audition. Philos. Trans. R. Soc. Lond. B Biol. Sci. 369:20130214. 10.1098/rstb.2013.0214 24639585 PMC3965168

[B58] Vorberg D. Schwarz W. (1987). Oscillatory mechanisms in human reaction times? Naturwissenschaften 74, 446–447. 10.1007/bf00446104 3683590

[B59] Waskom M. L. Kiani R. Okazawa G. Sha L. Purcell B. A. Kiani R. (2018). Decision making through integration of sensory evidence at prolonged timescales. Curr. Biol. 28, 3850.9–3856.9. 10.1016/j.cub.2018.10.021 30471996 PMC6279571

[B60] Wilsch A. Neuling T. Obleser J. Herrmann C. S. (2018). Transcranial alternating current stimulation with speech envelopes modulates speech comprehension. Neuroimage 172, 766–774. 10.1016/j.neuroimage.2018.01.038 29355765

[B61] Wostmann M. Herrmann B. Maess B. Obleser J. (2016). Spatiotemporal dynamics of auditory attention synchronize with speech. Proc. Natl. Acad. Sci. U S A 113, 3873–3878. 10.1073/pnas.1523357113 27001861 PMC4833226

[B62] Wyart V. de Gardelle V. Scholl J. Summerfield C. (2012). Rhythmic fluctuations in evidence accumulation during decision making in the human brain. Neuron 76, 847–858. 10.1016/j.neuron.2012.09.015 23177968 PMC3975574

[B63] Yi H. G. Leonard M. K. Chang E. F. (2019). The encoding of speech sounds in the superior temporal gyrus. Neuron 102, 1096–1110. 10.1016/j.neuron.2019.04.023 31220442 PMC6602075

[B64] Zoefel B. Davis M. H. (2017). Transcranial electric stimulation for the investigation of speech perception and comprehension. Lang. Cogn. Neurosci. 32, 910–923. 10.1080/23273798.2016.1247970 28670598 PMC5470108

[B67] Zoefel B. Davis M. H. Riecke L. (2019). How to test for oscillatory modulation of neural function and behaviour. bioRxiv [Preprint]. 10.1101/517243

[B68] Zoefel B. Reddy Pasham N. Bruers S. VanRullen R. (2015). The ability of the auditory system to cope with temporal subsampling depends on the hierarchical level of processing. Neuroreport 26, 773–778. 10.1097/wnr.0000000000000422 26164609

[B65] Zoefel B. VanRullen R. (2015). The role of high-level processes for oscillatory phase entrainment to speech sound. Front. Hum. Neurosci. 9:651. 10.3389/fnhum.2015.00651 26696863 PMC4667100

[B66] Zoefel B. VanRullen R. (2017). Oscillatory mechanisms of stimulus processing and selection in the visual and auditory systems: state-of-the-art, speculations and suggestions. Front. Neurosci. 11:296. 10.3389/fnins.2017.00296 28603483 PMC5445505

